# HoPE: Horizontal Plane Extractor for Cluttered 3D Scenes

**DOI:** 10.3390/s18103214

**Published:** 2018-09-23

**Authors:** Zhipeng Dong, Yi Gao, Jinfeng Zhang, Yunhui Yan, Xin Wang, Fei Chen

**Affiliations:** 1 School of Mechanical Engineering and Automation, Northeastern University, NO. 3-11, Wenhua Road, Heping District, Shenyang 110819, China; zhipengdongneu@gmail.com (Z.D.); yigaoyi@stumail.neu.edu.cn (Y.G.); jinfengzhang@stumail.neu.edu.cn (J.Z.); yanyh@mail.neu.edu.cn (Y.Y.); 2 Shenzhen Academy of Aerospace Technology, Shenzhen 100080, China; xinwanghit07s@gmail.com; 3 Department of Advanced Robotics, Istituto Italiano di Tecnologia, Via Morego 30, 16163 Genova, Italy

**Keywords:** 3D data segmentation, 3D imaging sensor, 3D point cloud, horizontal plane extraction, plane segmentation

## Abstract

Extracting horizontal planes in heavily cluttered three-dimensional (3D) scenes is an essential procedure for many robotic applications. Aiming at the limitations of general plane segmentation methods on this subject, we present HoPE, a Horizontal Plane Extractor that is able to extract multiple horizontal planes in cluttered scenes with both organized and unorganized 3D point clouds. It transforms the source point cloud in the first stage to the reference coordinate frame using the sensor orientation acquired either by pre-calibration or an inertial measurement unit, thereby leveraging the inner structure of the transformed point cloud to ease the subsequent processes that use two concise thresholds for producing the results. A revised region growing algorithm named Z clustering and a principal component analysis (PCA)-based approach are presented for point clustering and refinement, respectively. Furthermore, we provide a nearest neighbor plane matching (NNPM) strategy to preserve the identities of extracted planes across successive sequences. Qualitative and quantitative evaluations of both real and synthetic scenes demonstrate that our approach outperforms several state-of-the-art methods under challenging circumstances, in terms of robustness to clutter, accuracy, and efficiency. We make our algorithm an off-the-shelf toolbox which is publicly available.

## 1. Introduction

Modeling and understanding three-dimensional (3D) scenes have been hot research topics in the computer vision and robotics communities. With a variety of 3D sensors, such as 3D LiDARs, stereo cameras, or RGB-D cameras, robots can efficiently model 3D point clouds in real time. Within that, horizontal planes are of particular importance, since they can provide a rich structural description of the scene, represent the abundant supporting surfaces in human-made environments, and in some cases be the targets or cues for autonomous agents to perform particular tasks, including object extraction [1], simultaneous localization and mapping (SLAM) [2,3], collision-free motion planning [4,5], as well as tasks involving ground segmentation, such as autopilot [6] and assisting impaired people [7].

**Figure 1:**
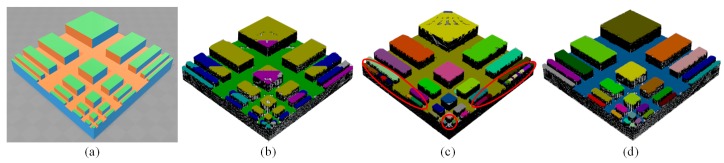
(**a**) A synthetic scene in Gazebo where a pattern object (detailed described in Section 3.3) was perceived by a simulated RGB-D sensor, yielding the point cloud to whom three plane extraction methods were applied, as depicted in (**b**–**d**). (**b**) Sloped results produced by RANSAC, note that points labeled with the same color belong to the same plane. (**c**) Under- and over-segmented results (marked with red ellipses) produced by Region Growing method. (**d**) Results produced by the proposed method. Best viewed in online color version.

However, semantically reconstruct the horizontal planes in a cluttered scene, wherein the structures being occluded, mixed with complicated background, or adjacent with each other [8], is not straightforward. In addition, it introduces five difficulties in such scenario for conventional methods dealing with plane segmentation:

1. It is hard to distinguish points of a plane from outliers belonging to the objects atop the plane or proximal planes of similar height. Fitting such points to a global model as done by RANSAC [9,10,11], Hough Transform (HT) [12,13] and Expectation-Maximization (EM) methods [14] commonly leads to producing sloped planes as depicted in Figure 1b, which is counter-factual regarding extracting horizontal planes and also complicates the computation of robotic tasks involving the surface’s pose, such as retrieving the objects upon the surface, determining where to step on during stair climbing or orienting the end-effector for picking or placing objects.
2. The results may be under- or over-segmented as depicted in Figure 1c. These phenomena are common for bottom-up methods such as Region Growing (RG) [15,16], of which a set of thresholds fails to find a balance between separating and merging the patches simultaneously. In addition, they give no clear instruction for relating quite a few thresholds with the output expected, such that the user can only determine that experimentally through an exhaustive search.
3. The detection can be computational expensive. Virtually, due to the existence of outliers and the difficulty of choosing thresholds, it is hard to reach the optimal without using time-consuming stabilizing methods.
4. It is hard to deal with both organized and unorganized point clouds. Line-segment-based plane segmentation methods [17,18] that address the challenge of efficiency can only deal with organized data, impairing their usage in unorganized case.
5. It is hard to preserve the identities (IDs) of the plane patches extracted among successive sequences as the robot moving around and changing its viewpoints. Robotic tasks usually refer a particular plane at a time, and it should not be confused with others. Nevertheless, such dynamic motion and the occlusion of objects within the scene damp the geometric characters of plane patches crucial for retaining the temporal consistency of the IDs. Conventional techniques such as SLAM [19] and Iterative Closest Point (ICP) [20,21] can address the problem, however, they are ponderous for matching plane patches, because we merely consider preserving the identities of planes instead of points.

Aiming at the limitations of conventional methods, we present HoPE (Horizontal Plane Extractor), a solution that robustly yet rapidly extracts multiple horizontal planes from both organized and unorganized 3D point clouds of cluttered scenes, and at the same time preserves the identities of each plane segments in successive frames.

The whole framework of HoPE takes full advantage of the sensor orientation prior in the first place. It transforms (if necessary) the source point cloud into reference frame $F_{r}$ whose z-axis points upwards, and regards the unit normal vector of all transformed horizontal surfaces denoted as n to be ideally perpendicular to the physical ground, such that n=(0,0,1) w.r.t. $F_{r}$ after transformation.

Basing on the assumption about n, we extract the inliers of horizontal planes whose normals fall within an error cone and then cluster them using a strategy named Z clustering, a revised RG approach that uses distances of neighbor points along the *z*-axis instead of local similarity of normals or curvatures as the growing criterion, which makes it more efficient and controllable with intuitive thresholds (detailed explained in Section 2.3). We apply the clustering to all *inliers* solely yet globally, such that it will not be distracted by extraneous points from non-horizontal regions, and hence remedis the under- or over-segment problem.

Nevertheless, Z clustering may be indiscriminate to clusters having eligible normals but in fact not horizontal. To deal with that, we holistically rule out specific false positive clusters (curved and sloped surfaces) by refinement dividing the normals of each cluster into two groups, and using a principal component analysis (PCA)-based approach to make the judgment without new thresholds (detailed explained in Section 2.4). Indeed, the whole framework managed to deliver the final results with only two thresholds, i.e., the resolution in the xy plane and the resolution along the *z*-axis, respectively. We illustrate the output of refinement in Figure 1d.

Moreover, we managed to keep the identity of each resulting plane in successive 3D sequences, regardless the changing of viewpoints and vibration. Generally, identifying multiple extracted planes is a non-trivial multi-object tracking problem, which requires robust features for identifing each object [22,23], especially when the movement of the sensor relevant to the reference coordinate frame is unknown. To address that, we seize the fact that the plane segments’ heights and point distribution change smoothly in continues 3D sequences. We demonstrated that with these constraints, the IDs could be preserved with a strategy using nearest neighbor algorithm as explained in Section 2.5, with consideration of target missing, heavy clutter and data noise.

It is worth pointing out that by its nature, our approach can only be directly applied to horizontal planes, whose normal directions are perpendicular with the ground, or in other words, parallel with the gravity direction. This criterion is required because in an unstructured environment, we can only make sure that the horizontal planes should be perpendicular with the ground, such that by aligning the z-axis of the reference frame $F_{r}$ with the gravity direction, we can check the error cone and clustering points using Z clustering. However, we usually have no prior knowledge of the pose of an arbitrary plane or even a vertical plane regarding $F_{r}$, which usually is identical with the robot’s base frame, and hence we cannot perform such alignment. However, if we do know the relative pose of the target plane within the reference frame, we can redirect the z-axis of $F_{r}$ to be vertical with that plane, and then using our approach along that direction to extract such targets.

In summary, our work makes the following contributions:

- Simplify the procedure of horizontal plane extraction with a sensor orientation guided transformation of 3D point clouds, providing approaches for fast yet robust clustering, refinement and identification which take full advantage of the inner structure of transformed point clouds.
- Minimize the number of thresholds used in a reasonable way, enabling the user to have a full control of the results in terms of the accuracy and computing time expected.
- An open-source horizontal plane extractor compatible with Point Cloud Library (PCL) [24] and Robot Operating System (ROS). It is available at https://github.com/DrawZeroPoint/hope.

## 2. Proposed Methodology

**Figure 2:**
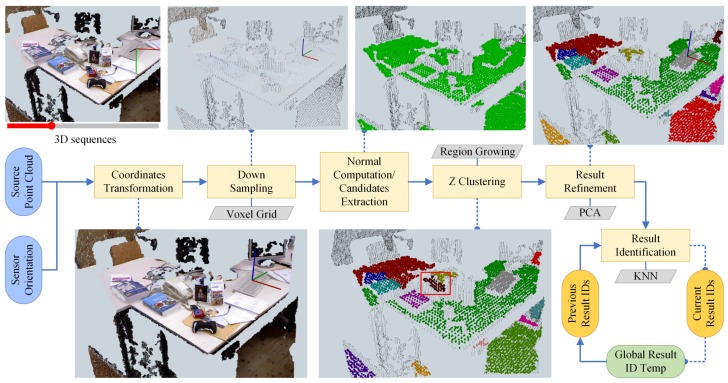
The architecture of the horizontal plane extraction pipeline along with the resulting screenshot of each procedure. The framework takes as input each point cloud in the 3D sequence and sensor orientation corresponding with that, which go through subsequential processing components. Please note that in the lower middle subfigure the area marked with red box indicated a slant surface of a telephone, which should not be included in the final results representing horizontal planes and was successfully filtered out by the result refinement method, as shown in the upper right subfigure. Best viewed in online color version.

An overview of our horizontal plane extractor (HoPE) is depicted in Figure 2 and detailed in the followings.

### 2.1. Input Data

The HoPE framework takes as input the orientations of the 3D sensor during data acquisition and the 3D point clouds generated. They can be received in real time by subscribing to corresponding ROS topics or automatically loaded from datasets for evaluation. The sensor orientations are used for transforming the source point clouds generated in the sensor’s coordinate frame $F_{s}$ into the reference frame $F_{r}$, whose xy plane is parallel to the ground and *z*-axis points upwards. The reference frame can either be fixed with the robot or with the physical world. Both configurations will make no difference to the results cause the translation is unnecessary in the framework, whereas it can be leveraged if the actual heights of planes are concerned. To get the relative pose between $F_{s}$ and $F_{r}$, we either compute it beforehand if that is fixed or obtain it in real-time by mounting an inertial measurement unit (IMU) on the sensor.

As for the 3D point cloud, we only assume that it can be processed to obtain the spatial position and normal orientation of points, whose color information is merely used for visualizing purpose. Hence it can be derived from various sources (e.g., 3D LiDAR, RGB-D camera, or stereo camera) in both organized and unorganized formats, for which the proposed method shows its flexibility and usability as indicated in Section 3. Since the RGB-D camera is one of the most used range sensors, we provide an algorithm in HoPE that generates point clouds using camera calibration parameters and synchronized image pairs obtained from the camera following the stereo registration paradigm.

### 2.2. Point Cloud Preprocessing

For each frame in the 3D data sequences, the source point cloud positioned in $F_{s}$ is firstly transformed into $F_{r}$ using corresponding sensor orientation. Let $p_{s}={\left(x_{s},y_{s},z_{s}\right)}^{T}$ be the point in $F_{s}$ and $p_{r}={\left(x_{r},y_{r},z_{r}\right)}^{T}$ the same point in $F_{r}$, the transformation from $p_{s}$ to $p_{r}$ can be expressed as:

(1) $$p_{r}=Rp_{s}+t$$

where R∈SO(3) is the rotation matrix, and $t\in R^{3}$ is the translation vector. Since the translation between the two frames is not used in our algorithm, we assume that $F_{r}$ has the same origin with $F_{s}$, so $t={\left(0,0,0\right)}^{T}$.

Typically, sensor orientations measured by human or an IMU are often expressed with Euler angles, i.e., roll, pitch, and yaw, which may cause ambiguity if the sets of rotation axes are not explicitly defined. To this end, we uniformly convert them into unit quaternion using the method provided by ROS tf2 package [25]. Besides, we do not restrict some of the Euler angles to be zero, extending the usability of the algorithm to a broader range. Please note that if the orientations of $F_{r}$ are the same with $F_{s}$, we omit to take them as input since the horizontal planes in the point cloud are already parallel to the ground as all we need.

With the relative orientation between $F_{s}$ and $F_{r}$ expressed in unit quaternion *Q* (i.e., xi+yj+kz+w=0), R is equal to:

(2) $$\left[\begin{array}{ccc} 1-2y^{2}-2z^{2} & 2xy+2wz & 2xz-2wy\\ 2xy-2wz & 1-2x^{2}-2z^{2} & 2yz+2wx\\ 2xz+2wy & 2yz-2wy & 1-2x^{2}-2y^{2} \end{array}\right]$$

After transforming the point cloud with Equations (1) and (2), the framework subsamples the transformed point cloud with the grid filter method implemented in PCL to efficiently reduce the noise in source data as well as the number of points to be processed. The grid dimensions of filtering are crucial parameters relating to both processing time and accuracy and influence subsequential procedures. We equate them with the resolution values (also serve as thresholds) of the proposed method, of which lower limits exist as the point cloud is discrete. Specifically, we equalize both the *x* dimension and *y* dimension of the grid with the resolution of the proposed method in xy plane w.r.t $F_{r}$ denoted as $r_{xy}$, and z dimension with the resolution along the *z*-axis of $F_{r}$ denoted as $r_{z}$.

With the filtered point cloud, the framework computes its normal denoted by the unit vector $n={\left(n_{x},n_{y},n_{z}\right)}^{T}$ with the method presented by [26], which requires a threshold on the radius for neighbor searching. For that, we set it to be $\delta \times r_{xy}$, where δ=1.01 in our configuration because δ only needs to be a constant larger than 1 to provide enough neighbor points for computing normal, whereas increasing it contributes less on the accuracy yet slow the process. A transformed point form the filterd point cloud is deemed to belong to horizontal planes if its normal satisfies the criterion:

(3) $$|n_{z}|>\frac{1}{\sqrt{1+2\gamma^{2}}}$$

where $\gamma =r_{z}/r_{xy}$. Notice that here we use the absolute value of $n_{z}$ because the normal computed by [26] can be opposite to the *z*-axis of $F_{r}$.

### 2.3. Z Clustering

With the candidate points derived from Equation (3), we cluster them into horizontal planes with a revised region growing method named Z clustering, which inherits the overall structure of RG [15] but differs from that regarding the clustering criterion used. Although we can directly employ [15] to cluster the candidates, it can be under perfect cause that requires setting the thresholds to evaluate the similarity of normals or curvatures, which are geometrically hard to formulate using $r_{xy}$ and $r_{z}$, and violates our motivation to produce the results by leveraging intuitional yet minimal amount of thresholds. Moreover, since the globally extracted candidates already have similar normal orientations, it is redundant and less efficient to use normals again as a criterion of RG, while using curvatures requires other time-consuming computation. Instead, we chose to leverage the distances of candidates along the *z*-axis of $F_{r}$ as the similarity criterion, letting the threshold for Z clustering equivalent to $r_{z}$, and taking the advantage that the *z* values of the transformed candidate points discriminatively represent the horizontal planes they belong. As for neighbor size of Z clustering, we check up to 8 neighbors for each candidate—since the point cloud is transformed and then filtered, an arbitrary candidate point within that and its neighbors tend to lie on the same plane or adjacent planes, thus with Z clustering the planes are more likely to extend horizontally but not vertically, which is precisely the property we demand.

### 2.4. Refinement with PCA

**Figure 3:**
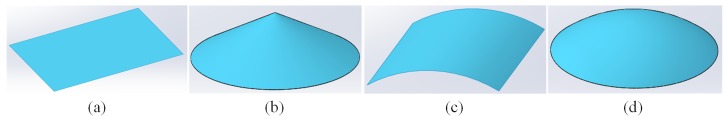
An illustration of typical geometric primitives that may result in false positives yielded by Z clustering. (**a**) A sloped plane whose gradient exceeds the threshold. (**b**) A cone. (**c**) The upper surface of a tube. (**d**) The upper surface of a sphere.

Although Z clustering endows HoPE with the ability of clustering plane point candidates with only two thresholds, the resulting clusters may contain some false positives as depicted in Figure 3, which may also fulfill the normal criterion and proximity criterion on both vertical and horizontal directions in a local perspective.

**Figure 4:**
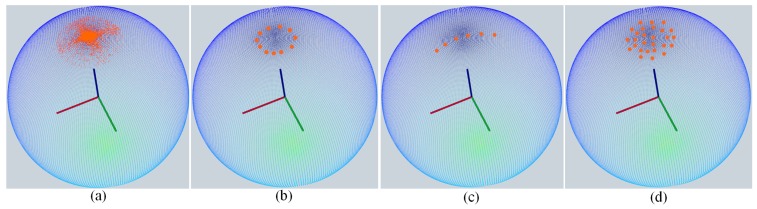
The Normal distributions on a normal sphere of different kinds of clusters after filtering with (3). (**a**) A horizontal plane, whose normals tend to obey a Gaussian distribution. (**b**) The normal distribution of a cone’s surface. (**c**) The normal distribution of a tube’s upper surface. (**d**) The normals of a sphere’s upper surface tend to follow a uniform distribution. Please note that only (**a**) is derived from real data, whereas the rest are synthesized for illustration purpose.

To tackle this issue, we propose a novel method which divides the normals from a cluster into two groups with the help of PCA and determines the horizontalness of clusters by comparing the *included angle* of the two mean normal vectors obtained from these two groups. This scheme is inspired by the normal distribution of different kinds of clusters on a normal sphere (also known as Extended Gaussian Image (EGI) [26]) whose Euclidean coordinate frame is a translation of $F_{r}$. As depicted in Figure 4, the cases in Figure 3b–d have distinct normal distributions on the sphere compared with the horizontal case, whereas by dividing the normals into two groups, the distinction becomes more obvious and can be quantitatively evaluated.

For each cluster obtained by Z clustering, its normals compose a set $N=\{n^{i}:3\leq i\leq K\}$ (note that if i≤2 the cluster is omitted as it is not likely to form a plane), where $n^{i}={\left({n_{x}}^{i},{n_{y}}^{i},{n_{z}}^{i}\right)}^{T}$ is the normal of the *i*-th point in the cluster, and *K* is the number of all points in that cluster, we compute the mean of N as:

(4) $$n_{mean}={\left(\frac{1}{K}\sum_{i=1}^{K}n_{x}^{i},\frac{1}{K}\sum_{i=1}^{K}n_{y}^{i},\frac{1}{K}\sum_{i=1}^{K}n_{z}^{i}\right)}^{T}$$

We project N onto the xy plane of $F_{r}$, such that the PCA algorithm is applied on 2D set denoted as $N^{\prime }\left(n^{\prime }\right)$, where $n^{\prime }={\left(n_{x},n_{y}\right)}^{T}$. We omit *i* for simplicity. Then, the set $N^{\prime }$ is arranged as a 2×K data matrix $X=\{X_{2i}\}$, whose empirical mean along each row (j∈[1,2]) is calculated as:

(5) $$\mu_{j}=\frac{1}{n}\sum_{i=1}^{n}X_{ji}\;\mathrm{and}\;\mu =\left[\begin{array}{c} \mu_{1}\\ \mu_{2} \end{array}\right]$$

With Equation (5), we center the data by subtracting the empirical mean vector μ from each column of the data matrix X, yielding the mean-subtracted matrix B, whose empirical covariance matrix C is determined as:

(6) $$C=\frac{1}{K-1}BB^{T}$$

For C, its eigenvalues and eigenvectors can be respectively expressed with the diagonal matrix D and matrix V:

(7) $$D=\left[\begin{array}{cc} \lambda_{1} & 0\\ 0 & \lambda_{2} \end{array}\right]\;and\;V=\left[\begin{array}{cc} v_{1}^{x} & v_{2}^{x}\\ v_{1}^{y} & v_{2}^{y} \end{array}\right]$$

With Equation (7), we determine the basis vector $A_{0}$ that represents the orientation of the first principal component of $N^{\prime }$ as:

(8) $$A_{0}=\left\{\begin{array}{cc} (v_{1}^{x},v_{1}^{y}), & \lambda_{1}\geq \lambda_{2}\\ (v_{2}^{x},v_{2}^{y}), & \lambda_{1}<\lambda_{2} \end{array}\right.$$

The two group $G_{1}$ and $G_{2}$ are then formed as:

(9) $$\left\{\begin{array}{cc} n\in G_{1} & \;\mathrm{if}\;A_{0}\cdot n^{\prime \prime }>0\\ n\in G_{2}, & \mathrm{else} \end{array}\right.$$

where $n^{\prime \prime }$ is the *i*-th column of matrix B; n is the corresponding normal in N. We compute the mean normal of group $G_{1}$ and $G_{2}$ respectively denoted as $n_{mean}^{1}$ and $n_{mean}^{2}$ following the same scheme as Equation (4), accordingly, the cluster is considered as horizontal if it satisfies:

(10) $$\left\{\begin{array}{c} \langle n_{mean},z\rangle <\arctan\gamma \\ \langle n_{mean}^{1},n_{mean}^{2}\rangle <\arctan\gamma \end{array}\right.$$

where $z={\left(0,0,1\right)}^{T}$; 〈a,b〉 denotes the acute angle formed by arbitrary vector a and b.

### 2.5. Nearest Neighbor Plane Matching

We use *vector* in C++ to present the results, of whom each element contains a cluster that satisfies Equation (10). As for applications requiring a convincing location for putting objects on, the point clusters can be more favorable than convex hulls, since hulls may cover the unseen gaps between plane segments, whereas all points in the cluster are based on solid observations. Besides outputting the vector that contains point clouds, we also output another vector of whom each element is a *feature vector* composed of five parameters of a cluster, namely, mean z ($z_{mean}$), minimum x ($x_{min}$), minimum y ($y_{min}$), maximum x ($x_{max}$), and maximum y ($y_{max}$) of points in the cluster. Please note that we do not use *x* or *y* coordinate of the cluster’s centroid as a source of features since the plane patches may be separated or be combined across the frames and the centroid of patches are not likely to hold their position in xy plane. In contrast, the extreme values of coordinates are less changeable even when the patch is shrunk or expanded under the scope of the sensor due to dynamic motion. Although it is possible to add extra extremum into the feature vector, we for now insist on the features mentioned above to balance between effectiveness and efficiency. The order of pushing features into vector makes no influence to the identification, since we are not using individual feature but using features jointly to infer the planes.

A standard method applicable to this scheme is the nearest neighbors (NN) method. We use FLANN [27] implemented in OpenCV as backbone in this case. Given two sets of feature vectors (each corresponding to a cluster) obtained from successive 3D sequences denoted as $v_{p}$ and $v_{c}$, our goal is to find a mapping between each element in $v_{c}$ with one in $v_{p}$, such that the IDs of previously observed planes can be inherited by successive ones. Otherwise we assign new IDs to successors if they outnumber the former ones or be less coherent with them compared with their competitors.

To make the NN algorithm ready for matching the feature vectors in five-dimensional space, we need to notice that the features are of different magnitude. For example, $z_{mean}$ is usually less than 3 m in indoor environments, whereas the other features can be both positive or negative ranging from 0 to tens or even hundreds. This difference can result in that only the most significant feature decides the mapping. To avoid that, we normalize each kind of feature for successive 3D sequences. Particularly, for each feature among the five $(z_{m}$,$x_{min}$,$y_{min}$,$x_{max}$,$y_{max})$, its value in previous and current frames for all clusters are denoted as $f_{p}^{i}$ and $f_{c}^{j}$, respectively, where i∈[1,m], j∈[1,k]; *m* and *k* are respectively the size of $v_{p}$ and $v_{c}$. We normalize the values as:

(11) $$f_{mean}=\frac{\sum_{i=1}^{m}f_{p}^{i}+\sum_{j=1}^{k}f_{c}^{j}}{m+k}$$

(12) $$f_{std}=\sqrt{\frac{\sum_{i=1}^{m}{\left(f_{p}^{i}-f_{mean}\right)}^{2}+\sum_{j=1}^{k}(f_{c}^{j}-f_{mean}{)}^{2}}{m+k}}$$

(13) $$f_{pn}^{i}=\frac{f_{p}^{i}-f_{mean}}{f_{std}}$$

(14) $$f_{cn}^{j}=\frac{f_{c}^{j}-f_{mean}}{f_{std}}$$

where $f_{pn}^{i}$ and $f_{cn}^{j}$ are normalized feature values. The fundamental idea behind Equations (11)–(14) is normalizing the features of the same type for all the clusters in both previous and current results jointly, which is beneficial compared with normalizing the features separately for the two results because it makes the relative distance among features intact. With that, we can derive the normalized feature vectors and feed them into the NN algorithm where only one nearest neighbor of each feature vector is considered. If two or more feature vectors of current results are matched with the same feature vector in the previous results, we preserve the one with minimum L2 distance while assigning the rest with new IDs—a strategy that has been proved by the experiments to be helpful for minimizing the matching error. As for the initialization of this algorithm, we assign natural numbers starting from zero with the IDs according to the order of extracting planes in the first run.

## 3. Experimental Results and Evaluations

We have evaluated HoPE together with two state-of-the-art plane segmentation methods (RANSAC and RG) on multiple horizontal plane extraction scenarios with two popular datasets as well as a synthetic scene provided by us. For all of these baseline methods, we use corresponding implements in PCL because of their well-documented, influential, and open source nature. We run all evaluations in an Intel Xeon E3-1231v3 desktop computer with 32-GB RAM, whose operating system is Ubuntu 14.04 with ROS Indigo and PCL 1.7 installed. Our open-source implementation includes data processing tools, implementation of reference methods, the synthetic scene, and instructions to run the evaluations in all these datasets.

### 3.1. TUM RGB-D Dataset

The TUM RGB-D dataset [28] contains highly cluttered indoors sequences from RGB-D sensors grouped in several categories. Some of the those are very challenging because they exhibit heavy clutter, dynamic motions, different textures, and varying illumination conditions. We used it and the parameters listed in Table 1 to perform evaluations described in this session.

**Figure 5:**
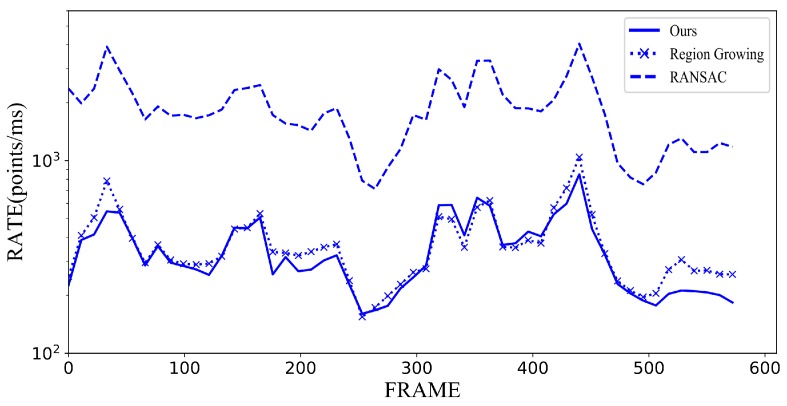
Runtime comparison of Hope and state-of-the-art on the *freburg1_desk* sequence. All runtime of three methods depends on the complexity of the scene. Despite that our approach involves extra result refining and identification procedures, our performance is compatible with original RG approach.

**Figure 6:**
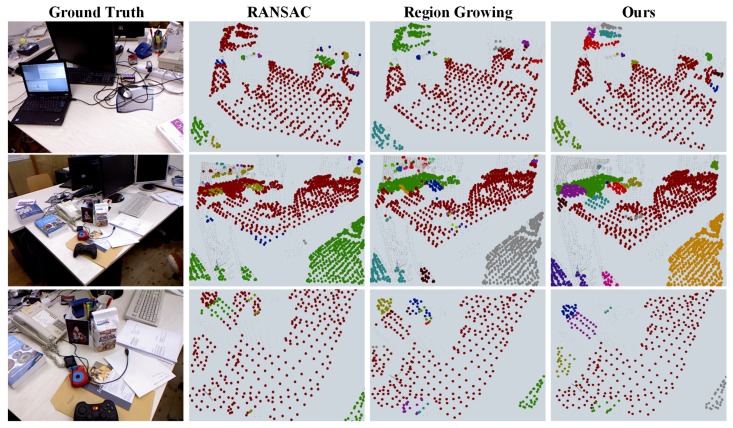
Intermediate results yielded by baseline methods and our methods on segmenting multiple horizontal planes in the 3D sequence *freburg1_desk* of TUM RGB-D dataset. Please note that our method is able to distinguish adjacent planes with similar height, as well as avoiding gently sloped planes such as the surface of the telephone in the second and the third columns. Best viewed in online color version.

In Figure 5, we compare our efficiency regarding processing *organized* point clouds (the 3D sequence is captured sequentially without merging) to aforementioned state-of-the-art methods. To make a fair comparison, we subsampled the source point cloud with the same thresholds for all methods. Meanwhile, the time consumed by preprocessing (subsampling, coordinate transformation, computing normal, and filtering) was not included. Hereby we show intermediate results in a subset (*freburg1_desk*) of the dataset on which most plane segmentation methods have been evaluated. As depicted in Figure 5 and Figure 6, our approach is of same order of magnitude with other methods in terms of efficiency yet outperforms the others or acquires similar results concerning accuracy in most sequences. We use the color table borrowed from Pascal VOC [29] to indicate the identities of the results.

Moreover, the identification capacity of our approach has been quantitatively evaluated on an expanded subset including *freburg1_desk*, *freburg1_xyz*, and *freburg1_rpy*. For that, we recorded the output of our method in video and manually accumulated true and false matches between successive 3D sequences. A correct mapping indicates that the same plane is labeled with the identical ID in the consecutive sequence, whereas a false one is on the contrary. Please note that if a single surface is separated into multiple parts during clustering, these parts should have different IDs and also considered separately. The accuracy ratio of the matches for the chosen subsets in TUM dataset is shown in Table 2.

### 3.2. Indoor LiDAR-RGBD Scan Dataset

**Figure 7:**
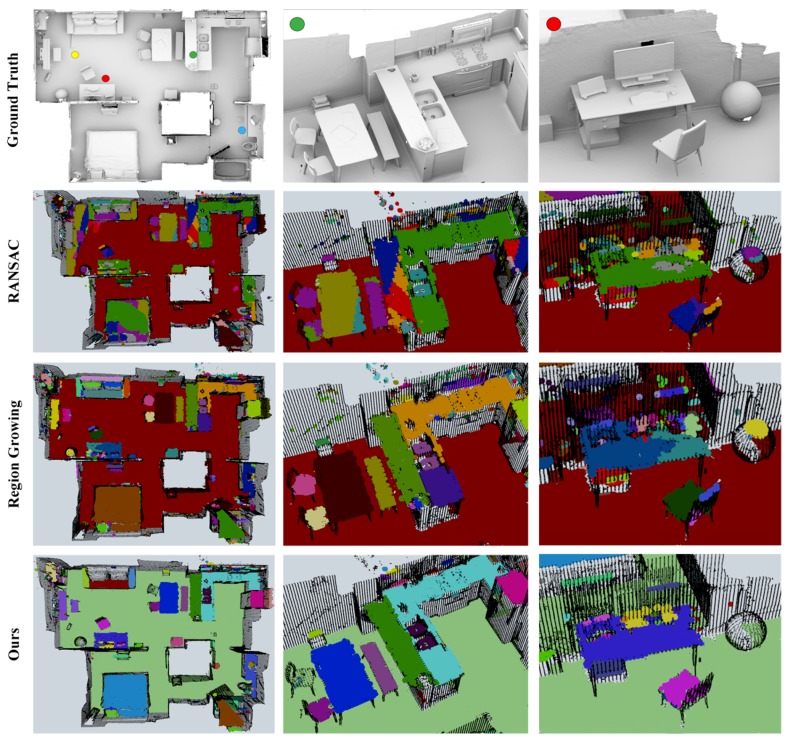
Intermediate results of the apartment scene from Indoor LiDAR-RGBD Scan Dataset. The parameters used in this experiment is detailed in Table 3. The baseline methods suffer from under- and over-segmentation especially for larger surfaces, which are avoided by the proposed method. Meanwhile, the curved surface such as the top surface of the sphere in the third column is successfully filtered out by our refinement method.

The Indoor LiDAR-RGBD Scan dataset [30] presents five large-scale real-world scenes (*apartment*, *bedroom*, *boardroom*, *lobby*, and *loft*) with ground-truth models and aligned point clouds, which can be used to benchmark the capacity of the plane segmentation methods dealing with substantial *unorganized* point clouds. We compare our runtime with baseline methods in Table 3. For this comparison, we run each scene five times and show averaged results as well as the standard deviations in order to account for the nondeterministic nature of the multithreading system, by which we confirmed that our method is slightly slower than RG knowing that the refining consumes a portion of time, whereas we outperform RANSAC by a large margin. The visual results of the apartment scene are illustrated in Figure 7. Please note that for each scene the coordinate frame wherein the point cloud positioned was aligned with $F_{r}$ using CloudCompare beforehand.

### 3.3. Synthetic Scene

**Figure 8:**
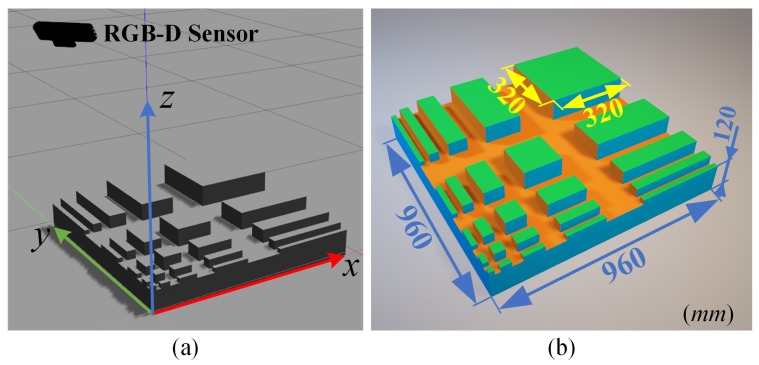
(**a**) The synthesized scene in Gazebo, where the pattern object is perceived by a simulated RGB-D sensor. The edges of the object’s base are respectively aligned with the *x* and *y* axes of the reference frame, while the edge orthogonal to those is aligned with the *z* axis. (**b**) The geometric features of the pattern object.

We have established a synthetic scene in Gazebo simulator [14] as shown in Figure 8a to simulate the percepts of a dedicated pattern object using an RGB-D sensor. We designed the object as a simulation of cluttered scene where planes can be multi-scale, slightly slant, and adjacent with each other. As shown in Figure 8b, the object is a platform holding cubes and cuboids of different sizes, whose top faces are horizontal to the base. Notably, the cubes’ dimensions in both *x* and *y* directions are $20\times 2^{n}$ mm, where n∈[0,4]. In addition, the height of these cubes is designed as 20×(n+1) mm. These cubes are located on one diagonal of the platform whose dimensions are 960 mm × 960 mm × 100 mm, and the cuboids are symmetrically located on both sides of the diagonal, whose dimensions are detailed in our open-source model. With this pattern object, we can evaluate horizontal plane segmentation methods regarding their robustness, effectiveness, and efficiency by extracting the top surfaces of these cuboids. The mildly raised top surfaces exam the capacity of providing non-slant planes; the gaps between the cuboids and the small surfaces act as a touchstone of avoiding under-segmentation, and the large surfaces testify the capability of avoiding over-segmentation.

**Figure 9:**
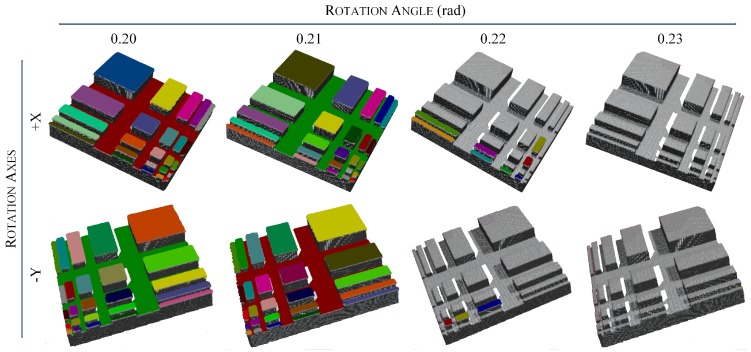
Results of the proposed method extracting multiple horizontal planes in the synthetic scene. Due to the thresholds configuration, planes with tilt angle less than 0.2187 rad were still considered as horizontal, yielding the extraction results as shown in the left two columns. When the tilt angle was close to the threshold as depicted in the third column, some small patches became false positive since their normals are sparser yet more sensitive to data noise. Nevertheless, the unstable state caused by noise disappeared as the tilt angle continue to increase 0.01 rad as shown in the rightmost column. Best viewed in online color version.

We exploited the synthetic scene to validate that adjusting the thresholds of the proposed method has a direct and intuitive impact of the discernibility as we assumed by covering a quantitative analysis of the effects. Notably, we choose the threshold for the evaluation to be $r_{xy}=0.009$ and $r_{z}=0.002$, such that the theoretical maximum slope angle is $\arctan(r_{z}/r_{xy})\approx 0.2187$ rad. In the experiment, we set the tilt angles of the object along +x and -y axes (the plus sign means rotating clockwise when looking along the axis, while the minus sign refers to counterclockwise rotation) of $F_{r}$ ranging from 0.2 rad to 0.23 rad with 0.01 rad increment, respectively. The results shown in Figure 9 imply that the proposed result refining method eliminates sloped plane segments with an error less than 0.01 rad. By this means, it is confirmed that our method is robust in such scenario, and the thresholds intuitively affect the results.

## 4. Conclusions

In this paper, we have presented a framework for extracting multiple horizontal planes from both organized and unorganized 3D point clouds acquired in clutter scenes. To fully exploit the inner structure of the point cloud to simplify procedures including subsampling, clustering, result refining, and result identification, our algorithm uses prior knowledge of sensor orientation in the first stage to transform the source point cloud into reference frame, whose *z*-axis points upwards. With the proposed framework composed of a series of dedicated and novel functions, we can deliver the results in a robust yet efficient way.

The potential advantages of our framework, in addition to the robustness and efficiency, are its scalability to the size of the scene and its ability to provide a continuous identification of the extracted results. Experiments on real datasets indicate that our method can keep the identities of the results even the scene exhibits a highly dynamic motion. Besides, tests on synthetic scene prove that our algorithm can tackle multiple horizontal planes of varying size with straight thresholds. By using the proposed method, we could provide reliable, temporally continuous horizontal surfaces with certainty for robotic and computer vision applications, and also applications involving horizontal plane extraction in cluttered 3D scenes.

## Figures and Tables

**Table 1:** Parameter Used for Evaluations on TUM Dataset.

Method	Parameter Except $r_{\mathrm{xy}}$ and $r_{z}$
RANSAC	Max iteration: 500
	Distance threshold: $\mu \cdot r_{xy}$
Region Growing	Number of neighbors (K): 20
	Smooth threshold: 8.0
	Curvature threshold: 1.0
Ours	-

**Table 2:** Identification on TUM Dataset.

Subset	Parameters Used for All Subsets	Accuracy (%)
*freburg1_360*		83.28
*freburg1_desk*	$r_{xy}=0.05$	82.99
*freburg1_rpy*	$r_{z}=0.01$	79.66
*freburg1_xyz*		84.33

**Table 3:** Runtime in LiDAR-RGBD Scan Dataset (Points/ms).

Scene	Point Number	RANSAC	RG	Ours
*apartment*	160,554	446±1	966±15	891±2
*bedroom*	118,991	804±5	1279±6	1226±5
*boardroom*	180,986	545±3	796±16	825±5
*lobby*	313,960	215±1	755±9	859±2
*loft*	144,011	384±1	1121±10	879±3

## References

[1] Ecins A., Fermüller C., Aloimonos Y. Cluttered scene segmentation using the symmetry constraint. Proceedings of the 2016 IEEE International Conference on Robotics and Automation (ICRA 2016).

[2] Cho H., Yeon S., Choi H., Doh N. (2018). Detection and Compensation of Degeneracy Cases for IMU-Kinect Integrated Continuous SLAM with Plane Features. Sensors.

[3] Trevor A.J.B., Rogers J.G., Christensen H.I. Planar surface SLAM with 3D and 2D sensors. Proceedings of the 2012 IEEE International Conference on Robotics and Automation (ICRA 2012).

[4] Farid R., Pfahringer B., Renz J. (2015). Region-Growing Planar Segmentation for Robot Action Planning. AI 2015: Advances in Artificial Intelligence.

[5] Zhang T., Caron S., Nakamura Y. (2017). Supervoxel Plane Segmentation and Multi-Contact Motion Generation for Humanoid Stair Climbing. Int. J. Hum. Robot..

[6] Dos Santos G.A.M., Ferrão V.T., Vinhal C.D.N., da Cruz Junior G. (2016). Fast algorithm for real-time ground extraction from unorganized stereo point clouds. Pattern Recogn. Lett..

[7] Herghelegiu P., Burlacu A., Caraiman S. Robust ground plane detection and tracking in stereo sequences using camera orientation. Proceedings of the 2016 20th International Conference on System Theory, Control and Computing (ICSTCC 2016).

[8] Teng Z., Xiao J. (2016). Surface-Based Detection and 6-DoF Pose Estimation of 3-D Objects in Cluttered Scenes. IEEE Trans. Robot..

[9] Fischler M.A., Bolles R.C. (1981). Random Sample Consensus: A Paradigm for Model Fitting with Applications to Image Analysis and Automated Cartography. Commun. ACM.

[10] Gallo O., Manduchi R., Rafii A. (2011). CC-RANSAC: Fitting planes in the presence of multiple surfaces in range data. Pattern Recogn. Lett..

[11] Qian X., Ye C. (2014). NCC-RANSAC: A Fast Plane Extraction Method for 3-D Range Data Segmentation. IEEE Trans. Cybernet..

[12] Vera E., Lucio D., Fernandes L.A., Velho L. (2018). Hough Transform for real-time plane detection in depth images. Pattern Recogn. Lett..

[13] Limberger F.A., Oliveira M.M. (2015). Real-time detection of planar regions in unorganized point clouds. Pattern Recogn..

[14] Thrun S., Martin C., Liu Y., Hahnel D., Emery-Montemerlo R., Chakrabarti D., Burgard W. (2004). A real-time expectation-maximization algorithm for acquiring multiplanar maps of indoor environments with mobile robots. IEEE Trans. Robot. Autom..

[15] Rabbani T., van den Heuvel F., Vosselman G. (2006). Segmentation of point clouds using smoothness constraint. Int. Arch. Photogr. Remote Sens. Spat. Inf. Sci..

[16] Xiao J., Zhang J., Adler B., Zhang H., Zhang J. (2013). Three-dimensional point cloud plane segmentation in both structured and unstructured environments. Robot. Auton. Syst..

[17] Georgiev K., Creed R.T., Lakaemper R. Fast plane extraction in 3D range data based on line segments. Proceedings of the 2011 IEEE/RSJ International Conference on Intelligent Robots and Systems (IROS 2011).

[18] Pang C., Zhong X., Hu H., Tian J., Peng X., Zeng J. (2018). Adaptive Obstacle Detection for Mobile Robots in Urban Environments Using Downward-Looking 2D LiDAR. Sensors.

[19] Zhang L., Chen D., Liu W. Point-plane SLAM based on line-based plane segmentation approach. Proceedings of the 2016 IEEE International Conference on Robotics and Biomimetics (ROBIO 2016).

[20] Dubé R., Gawel A., Sommer H., Nieto J., Siegwart R., Cadena C. An online multi-robot SLAM system for 3D LiDARs. Proceedings of the 2017 IEEE/RSJ International Conference on Intelligent Robots and Systems (IROS 2017).

[21] Dubé R., Gollub M.G., Sommer H., Gilitschenski I., Siegwart R., Cadena C., Nieto J. (2018). Incremental-Segment-Based Localization in 3-D Point Clouds. IEEE Robot. Autom. Lett..

[22] Luan S., Chen C., Zhang B., Han J., Liu J. (2018). Gabor convolutional networks. IEEE Trans. Image Process..

[23] Zhang B., Gu J., Chen C., Han J., Su X., Cao X., Liu J. (2018). One-two-one networks for compression artifacts reduction in remote sensing. ISPRS J. Photogramm. Remote Sens..

[24] Rusu R.B., Cousins S. 3D is here: Point Cloud Library (PCL). Proceedings of the 2011 IEEE International Conference on Robotics and Automation (ICRA 2011).

[25] Foote T. tf: The transform library. Proceedings of the 2013 IEEE Conference on Technologies for Practical Robot Applications (TePRA 2013).

[26] Rusu R.B. (2010). Semantic 3D Object Maps for Everyday Manipulation in Human Living Environments. Künstliche Intell..

[27] Muja M., Lowe D.G. (2009). Fast approximate nearest neighbors with automatic algorithm configuration. VISAPP.

[28] Sturm J., Engelhard N., Endres F., Burgard W., Cremers D. A benchmark for the evaluation of RGB-D SLAM systems. Proceedings of the 2012 IEEE/RSJ International Conference on Intelligent Robots and Systems (IROS 2012).

[29] Everingham M., Van Gool L., Williams C.K., Winn J., Zisserman A. (2010). The Pascal Visual Object Classes (VOC) Challenge. Int. J. Comput. Vis..

[30] Park J., Zhou Q.Y., Koltun V. Colored Point Cloud Registration Revisited. Proceedings of the 2017 IEEE International Conference on Computer Vision (ICCV 2017).

